# Transcriptomic Signatures of Ash (*Fraxinus* spp.) Phloem

**DOI:** 10.1371/journal.pone.0016368

**Published:** 2011-01-21

**Authors:** Xiaodong Bai, Loren Rivera-Vega, Praveen Mamidala, Pierluigi Bonello, Daniel A. Herms, Omprakash Mittapalli

**Affiliations:** 1 Department of Entomology, The Ohio State University, Ohio Agricultural and Research Development Center, Wooster, Ohio, United States of America; 2 Department of Plant Pathology, The Ohio State University, Columbus, Ohio, United States of America; University of Minnesota, United States of America

## Abstract

**Background:**

Ash (*Fraxinus* spp.) is a dominant tree species throughout urban and forested landscapes of North America (NA). The rapid invasion of NA by emerald ash borer (*Agrilus planipennis*), a wood-boring beetle endemic to Eastern Asia, has resulted in the death of millions of ash trees and threatens billions more. Larvae feed primarily on phloem tissue, which girdles and kills the tree. While NA ash species including black (*F. nigra*), green (*F. pennsylvannica*) and white (*F. americana*) are highly susceptible, the Asian species Manchurian ash (*F. mandshurica*) is resistant to *A. planipennis* perhaps due to their co-evolutionary history. Little is known about the molecular genetics of ash. Hence, we undertook a functional genomics approach to identify the repertoire of genes expressed in ash phloem.

**Methodology and Principal Findings:**

Using 454 pyrosequencing we obtained 58,673 high quality ash sequences from pooled phloem samples of green, white, black, blue and Manchurian ash. Intriguingly, 45% of the deduced proteins were not significantly similar to any sequences in the GenBank non-redundant database. KEGG analysis of the ash sequences revealed a high occurrence of defense related genes. Expression analysis of early regulators potentially involved in plant defense (i.e. transcription factors, calcium dependent protein kinases and a lipoxygenase 3) revealed higher mRNA levels in resistant ash compared to susceptible ash species. Lastly, we predicted a total of 1,272 single nucleotide polymorphisms and 980 microsatellite loci, among which seven microsatellite loci showed polymorphism between different ash species.

**Conclusions and Significance:**

The current transcriptomic data provide an invaluable resource for understanding the genetic make-up of ash phloem, the target tissue of *A. planipennis*. These data along with future functional studies could lead to the identification/characterization of defense genes involved in resistance of ash to *A. planipennis*, and in future ash breeding programs for marker development.

## Introduction

Ash (*Fraxinus* spp.) is a dominant tree species in many urban and forest landscapes of North America (NA) [1], [2]. The emerald ash borer (*Agrilus planipennis* Fairmaire, EAB), which is indigenous to Eastern Asia has killed millions of ash trees since its accidental introduction to NA, primarily in the Midwestern United States and Southeastern Ontario [3], [4]. Larvae feed on phloem and outer xylem of trees of all sizes, girdling the tree and ultimately killing it within 1–4 years after symptoms become apparent [3], [4]. Black (*F. nigra* Marshall), green (*F. pennsylvanica* Marshall), and white ash (*F. americana* L.) are known to be highly susceptible, while blue ash (*F. quadrangulata* Michx) appears to be less preferred [5], [6]. If the pattern of invasion continues, *A. planipennis* has the potential to decimate ash throughout NA with substantial economic and ecological impact [3], [7]–[8].

Conversely, *A. planipennis* is not reported to be a major pest in Asia, where Manchurian ash (*F. mandshurica* Rupr) is a primary host [9]. In a common garden experiment, Manchurian ash was found to be much more resistant to *A. planipennis* than were NA green and white ash, perhaps by virtue of the co-evolutionary history shared by *A. planipennis* and Manchurian ash [10]. Phloem tissue of Manchurian ash was found to have high constitutive concentrations of phenolic-based hydroxycoumarins, phenylethanoids and calceloariosides, which may contribute to its resistance to *A. planipennis* [11].

Second generation sequencing technologies have been applied to a wide variety of studies such as transcriptome sequencing, single nucleotide polymorphism (SNP) discovery, mutation mapping, alternative splicing identification etc. [12]–[15]. In particular, gene discovery via transcriptome analysis has greatly helped in genomic analysis of several non-model organisms including plants viz., *Cucumis sativus* [16], *Eucalyptus grandis* [13], *Castanea dentate* and *C. mollisima* [17] and *Pinus contorta* [18]. Roche® 454 GS FLX Titanium is a high throughput sequencing platform that makes it possible to generate massive amounts of information in a short period of time with unprecedented high sequencing depth and low cost [19]. The generated expressed sequenced tags (ESTs) databases are invaluable for gene mining and annotation [20]–[24], phylogenetic analysis [25], molecular markers [26] and expression analysis [27].

Given the status of *A. planipennis* as an aggressive killer of NA ash trees, we undertook a functional genomics approach to identify the repertoire of genes expressed in phloem tissue of different ash species including green, white, black, blue, and Manchurian ash. This study will enable us to identify genes that are potentially involved in *A. planipennis* resistance of Manchurian ash, and to characterize the genetic makeup of ash phloem for future studies. Results stemming from this study could be used in future ash targeted breeding programs and increase fundamental understanding of interaction between ash trees and wood-borers such as *A. planipennis*.

## Results and Discussion

### Transcriptome analysis

**Figure 1:**
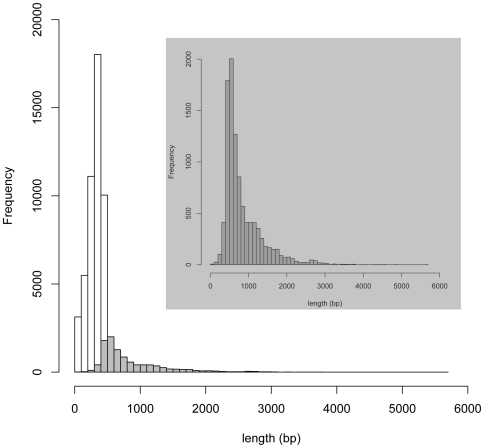
Summary of *Fraxinus* spp. transcriptomic sequences. The singleton sequences are represented by clear bars and the contig sequences by shaded bars (insert).

So far, one cDNA library has been developed for *F. excelsior* (European ash), and no EST sequences were available for NA and Manchurian ash as of October, 2010. The 454 pyrosequencing has made possible genomic studies in non-model organisms because it overcomes the limitations of conventional Sanger sequencing [18]. Our pyrosequencing study contributes a significant number of ESTs for future functional genomic studies in ash, yielding 203,718 total reads and 63,096,022 bases from which 79% and 60% were aligned respectively with an inferred read error of 2.21%. Assembled contigs had an average size of 649 bp with the largest contig being 5,662 bp. The singletons had an average size of 329 bp with the largest being 964 bp. Overall, we obtained 58,673 high quality ash sequences totaling 23,580,430 bp (Figure 1). To our knowledge, this is the first comprehensive study on the transcriptome of ash phloem.

**Figure 2:**
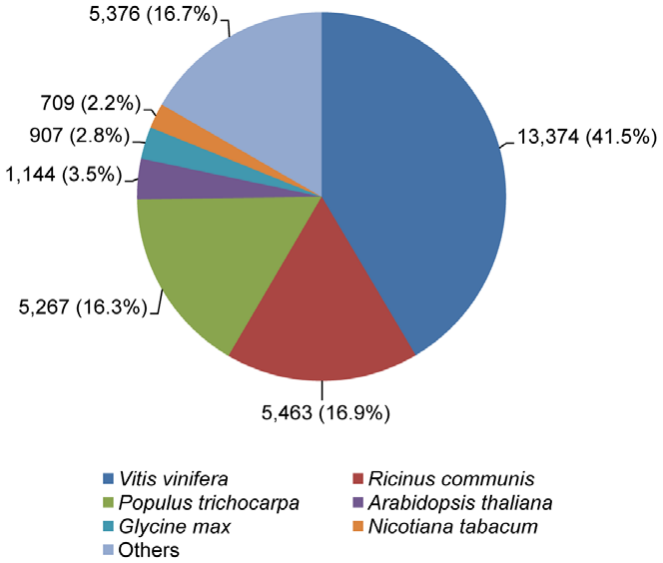
A pie chart showing species distribution of the top BLAST hits of the *Fraxinus* spp. sequences to various plant species.

A sequence similarity search was done using BLASTx algorithm. This analysis revealed that 45% of the ash transcriptomic sequences to be potentially ash-specific i.e., no significant matches (E value cutoff of 1e-5) to protein sequences in the GenBank nr database. The vast majority (>99%) of the sequences with significant matches matched to plant sequences (Figure 2), out of which 41.5% matched with *Vitis vinifera* L., 16.3% with *Populus trichocarpa* (Torr. & Gray) and 16.9% with *Ricinus communis* L. In our dataset, 9 sequences matched to viral sequences, 18 to artificial sequences, and 42 to bacterial sequences. Although some of these (viral, artificial and bacterial) sequences may be derived from organisms that are naturally associated with ash trees, they were excluded from further analysis due to possible contamination.

### Comparative analysis

**Figure 3:**
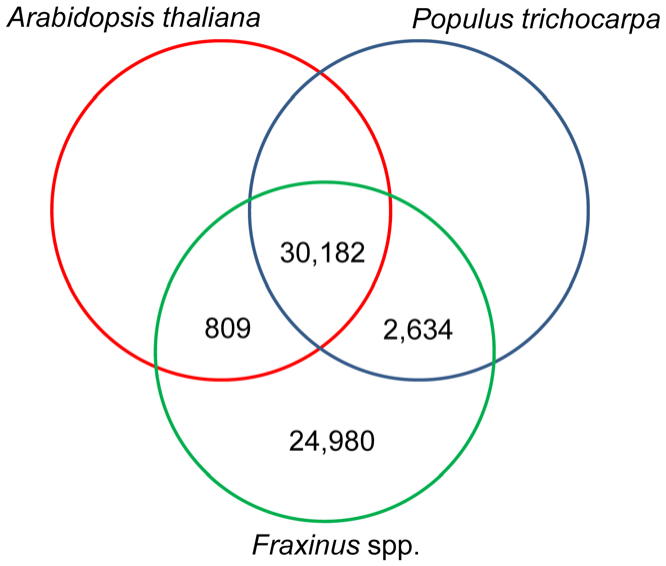
A venn diagram showing the comparisons of the sequences from *Fraxinus* spp. with the genomes sequences of *Arabidopsis thaliana* and *Populus trichocarpa*.

The ash transcriptomic sequences were compared to protein sequences of the model plant *Arabidopsis thaliana* (Brassicaceae) and the black cottonwood *P. trichocarpa* (Salicaceae) (also known as western balsam poplar or California poplar). These two species were chosen as they represent model plant systems whose genomes have been sequenced. Of the total 58,604 ash sequences, 24,980 (42.6%) had no significant similarity to any protein identified within the genomes of *A. thaliana* or *P. trichocarpa* (Figure 3). Similar observations of species specific transcript sequences were observed in other transcriptomic studies and were attributed to the presence of novel sequences or transcripts of 5′ and 3′ untranslated regions or genes with homologs in other species whose biological functions are not yet assigned. [28]–[30]. About 2,634 (4.5%) sequences were shared between *P. trichocarpa* and *Fraxinus* spp., but not with *A. thaliana*, suggesting that they are potential tree-specific sequences. Only 809 (1.4%) sequences were shared between *A. thaliana* and *Fraxinus* spp (Table S1). There were 30,182 sequences (51.5%) that were shared among all three plant species under comparison. Comparative genomics explore similarity with transcriptomes of other species, reveals species specific details and define genes which are conserved or diverging in plant species [31], [32]. Genome sequencing in non-model organisms, particularly forest trees is still in its infancy. For such species functional and comparative genomics is possible upon obtaining a good EST database. These studies seem to be the best source for deciphering the putative function of novel genes [33].

### Gene Ontology

**Figure 4:**
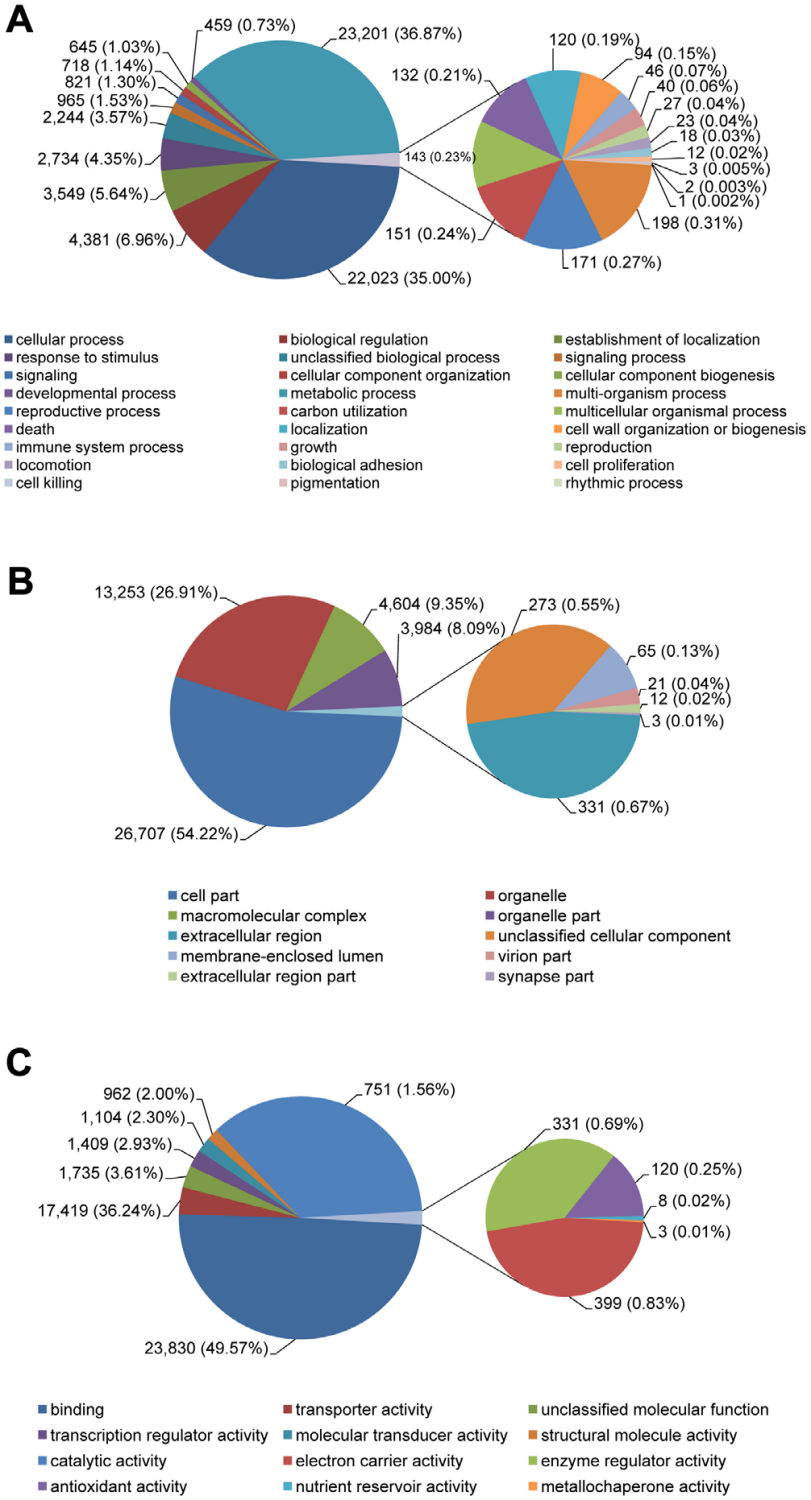
Depiction of Gene ontology (GO) terms for the transcriptomic sequences of *Fraxinus* spp. (A) Biological Process, (B) Cellular Component and (C) Molecular Function.

The derived *Fraxinus* phloem transcripts were assigned to three functional groups based on Gene Ontology (GO) terminology: Biological Process, Molecular Function and Cellular Component (Table S2). The software assigned 1,248 biological process terms to 19,958 transcripts (Figure 4A), 396 cellular component terms to 17,977 transcripts (Figure 4B), and 1,386 molecular function terms to 24,294 transcripts (Figure 4C). The most represented biological process terms were related to development (36.87%) and carbon utilization (35%). For those assigned to the cellular component, 54.22% were presumably associated with the cell part and 26.91% of the transcripts were representative for the organelle. Finally, the majority of terms represented in molecular function were for binding (49.57%) and catalytic activity (36.24%) suggesting a high degree of basal metabolic activity. It is well documented that a higher percentage of transcripts are involved in binding and catalytic activity in phloem compared to other parts of the plant [34]. We identified 417 *Fraxinus* transcripts encoding for proteins that are potentially involved in stress responses, including 120 transcripts encoding for heat-shock proteins. These proteins could be involved in responding to external stimuli including biotic and abiotic factors [35]. Functional annotation is a prerequisite to better understand transcriptomic data (especially of non-model systems). The GO facilitates functional characterization of genes, transcripts and proteins of any organisms with respect to cellular component, biological process and molecular function in a species-independent manner as reported in several other studies [36]–[38].

### Metabolic pathways

Overall 4,667 sequences were assigned to 142 KEGG metabolic pathways and the number of transcripts in the different pathways ranged from 1 to 1,422 (Table S3). The highest number of transcripts (1,422) was found in the secondary metabolites biosynthesis pathway (Table 1). In total, 788 transcripts that are involved in 7 biosynthesis pathways for alkaloids including indole alkaloid, isoquinoline alkaloid, tropane, piperidine and pyridine alkaloids were predicted in the ash sequences. Alkaloids are important components of the plant defense system against insect herbivory and so far 12,000 different alkaloids have been reported in plants which are involved in plant defense, growth and development [39], [40].

**Table 1:** Putative defense pathways identified in *Fraxinus* spp.

Pathway	# of ESTs
Biosynthesis of secondary metabolites	1422
Biosynthesis of plant hormones	668
Biosynthesis of phenylpropanoids	531
Thiamine metabolism	232
Arginine and proline metabolism	191
Cysteine and methionine metabolism	169
Valine, leucine and isoleucine degradation	151
Phenylalanine metabolism	127
Lysine degradation	123
Tryptophan metabolism	123
Tyrosine metabolism	112
Flavonoid biosynthesis	96
Drug metabolism (cyt P450)	93
Metabolism of xenobiotic by cyt P450	72
Anthocyanin biosynthesis	22
Isoflavonoid biosynthesis	10

In this study, we recovered a high number of transcripts (531) that were mapped to the phenylpropanoid biosynthesis pathway. This pathway leads to the production of several phenolic compounds (flavonoids, tannins, coumarins etc.,) that plays an important role in plant defense against herbivores, microbes, and wounding [41]–[44]. Although not all of the major genes reported in the pathway were found in this study, this information provides a good base for further analysis and to better understand the potential role of phenylpropanoids in ash defense against biotic stress.

### Protein domains

A domain search using HMMER3 software identified 2,534 distinct domains in 19,291 ash transcriptomic sequences (Table S4). Among the top Pfam domains, the most abundant ones were protein kinase domains (588) and protein tyrosine kinase domains (464). Protein kinases are primarily involved in plant signal transduction pathways [45], [46] and also participate in plant defense responses wherein they play an important role in signaling during pathogen recognition and activation of other plant defense mechanisms [47]–[49]. On the other hand, proteins containing tyrosine kinase domains and protein tyrosine phosphatases (PTPs) regulate abscisic acid (ABA) transduction pathways in plants [50]. The role of PTPs has been largely ignored; however a few tyrosine specific phosphatases were reported in *A. thaliana* [51]–[52]. PTPs are well documented in *Daucus carota, Mimosa pudica, Arabidopsis* hypocotyls and suspension cells [53]–[56]. Other abundant domains included metallothionein (263) and RNA recognition motif (RRM, 231). While metallothioneins are primarily involved in copper detoxification [57], RRM (also known as RNA binding domain or Ribonucleoprotein domain) plays an important role in post transcriptional events and in particular is involved in the 3′ end processing of chloroplast mRNA, [58], [59]. In a recent study, RRMs have emerged as key players in plant morphogenesis and RNA metabolism in chloroplast and mitochondria [60]. Further, we identified 154 RAS family members, which constitute RAS, RHO, RAB/YPT, ARF and RAN. RAS and RHO GTPases are considered to be important components in signaling cascades [61].

Interestingly, 153 cytochrome P450 domains were predicted in the derived ash sequences. Plant cytochrome P450 monoxygenases are thought to be involved in many biochemical pathways including the biosynthesis of secondary metabolites (e.g. phenylpropanoids, alkaloids, terpenoids, glucosinolates etc.), which have been well studied in plant-insect interactions [62]. However, plant cytochrome P450s are also involved in the biosynthesis of brassinosteroids and plant growth regulators [63], [64].

In total, 92 PPR (pentatricopeptide repeat) domains were identified in the ash sequences. The PPR repeat domain of ∼35 amino acids are well-known protein family members of both prokaryotes and eukaryotes [65] and appeared to function as sequence-specific RNA-binding proteins involved in post-transcriptional processes within organelles and translation initiation [66]–[71]. We also identified the PIWI domain in 18 transcripts and the PAZ domain in 6 transcripts. These domains are reported to be up regulated in the egg of *A. thaliana* and the presence of these domains suggests their role in epigenetic regulations through small RNA pathways [72].

### Genes of Interest

Plants being sessile overcome various biotic and abiotic stress conditions through controlled gene expression. Immediate recognition of the biotic or abiotic factors/stimuli (i.e. in the early stages) is one of the key factors in plant defense [73]. Of the potential genes of interest listed in Table 2, we are particularly interested in those genes that participate in the early stages of plant defense including calcium dependent protein kinases (CDPKs); the transcription factors (TFs) WRKYs, MYBs and ethylene response factor (ERF); and a lipoxygenase (LOX3).

**Table 2:** Genes of interest recovered from the *Fraxinus* spp. transcriptomic database.

Candidate genes	Number of occurrence
Proteases	282
cytochrome P450	192
Lipase	94
**WRKYS** *	**47**
**CDPKS**	**43**
**MYB**	**37**
Hydroxyproline-rich glycoprotein	29
Protease/proteinase inhibitors	16
Phytoalexin deficient 4 (PAD4)	11
Hypersensitive-induced response protein	9
DREB	07
Myrosinase	7
**Lipoxygenase**	**6**
Jasmonic acid-amino conjugating enzyme	3
Pathogen-related protein	3
**ERF**	**02**

*Candidate genes assayed in this study (in bold).

**Figure 5:**
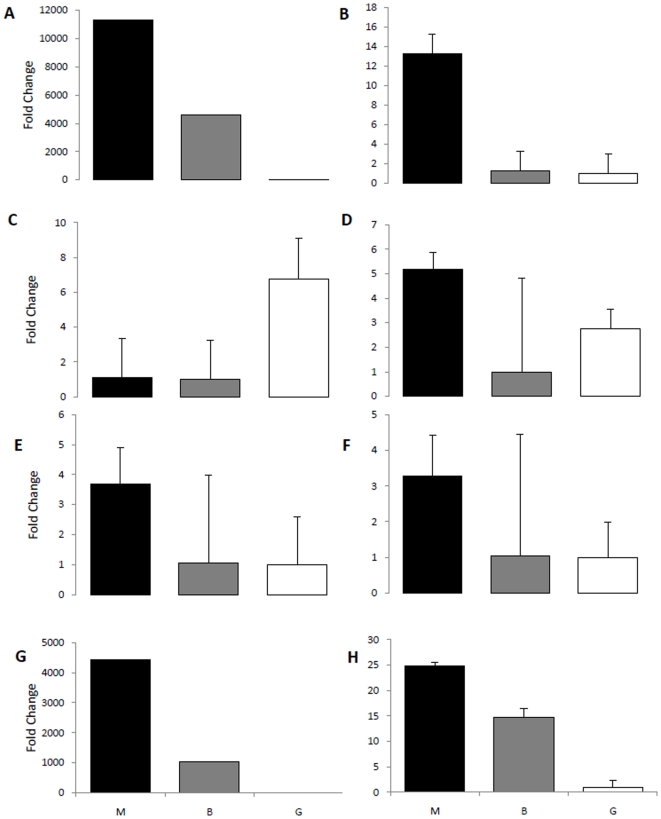
Quantitative PCR analysis of candidate early regulators in different *Fraxinus* spp. Includes Manchurian ash (M, black bars), black ash (B, grey bars) and green ash (G, white bars). Expression levels are shown for two CDPKs (A & B); two WRKYs (C & D), two MYBs (E & F), an ethylene response factor (G), and a lipoxygenase (H). An ash glucose-6-phosphate dehydrogenase (G6PD) was used as the internal reference gene. Standard error of the mean for two biological replicates (nested with two technical replicates) is represented by the error bars.

In the current study, both CDPKs (CDPK 349 and CDPK 361) showed the highest mRNA levels in Manchurian ash followed by black ash and green ash (Figure 5A and 5B). Interestingly, both CDPKs of ash significantly matched with CDPK3 of *Nicotiana tabacum* and *P. trichocarpa* (1e-14 and 6e-53). In a recent study, it was reported that CDPK3 and CDPK13 are involved in herbivory-induced signaling network via the regulation of defense related transcriptional machinery in *A. thaliana* [74]. Usually a dramatic change in cytosolic Ca^2+^ is observed through signaling pathways mediated by CDPKs in plants upon biotic and abiotic stress [75]–[77]. These findings along with the expression analysis could suggest that the recovered *Fraxinus* CDPKs (349 and 361) may regulate the transcriptional machinery involved in defense response. We posit that, the observed (constitutive) high mRNA levels for both CDPKs in Manchurian ash (compared to the susceptible black and green ash) may represent an enhanced capability to defend against *A. planipennis*. However, further studies need to be performed to confirm these observations.

Upon physiological and environmental stimuli TFs (sequence specific DNA binding proteins) modulate transcription of specific target genes by binding to *cis*-elements located in gene promoters and/or introns [78]–[80]. TFs represent potential candidate genes for developing novel traits in crop plants [81]. In the current transcriptomic study, we found high occurrence of WRKYs, which are key regulators in higher plants represent the top ten largest families of transcription factors and are found throughout the green plants [82]. These early regulators are involved in modulating defense responses, abiotic stress and biosynthesis of secondary metabolites [83]–[86]. Expression analysis of two WRKYs (WRKY 7 and WRKY21) in ash revealed higher mRNA levels for WRKY 7 in Manchurian ash and for WRKY21 in green ash compared to the other ash species assayed (Figure 5C and 5D). These results may suggest that the profiled WRKYs respond to different external stimuli including biotic and abiotic stress. As reviewed in many previous studies, overexpression of OsWRKY resulted in enhanced salt and drought tolerance and the AtWRKY 25 mutants exhibited increased thermosensitivity [82], [87]. Besides these biotic and abiotic stress responses, WRKY proteins are reported to be involved in sugar signaling and seed development [88]–[91].

Both the MYBs (MYB8679 and MYB10337) assayed in ash were highly expressed in Manchurian ash compared to the mRNA levels observed for green and black ash species (Figure 5E and 5F). MYBs are reported to be involved in several physiological and biochemical processes including defense and stress response, regulation of secondary metabolism and signaling pathways [92]–[96].

In the current study, we found an ethylene response factor (ERF) that showed higher mRNA levels in Manchurian ash compared to green and black ash (Figure 5G). ERFs are important TFs that bind to the GCC motif of promoter region of ethylene-regulated genes [97]. In response to insect attack, plants usually show an ethylene burst which eventually activates polyphenol oxidase, peroxidase and proteinase inhibitor activities [98]. Ethylene is known to regulate a large number of genes related to defensive proteins and secondary metabolites [99], [100]. In a recent study it was shown that several genes involved in ethylene signaling were upregulated during forest tent caterpillar (*Malacosma disstria*) feeding on hybrid poplar leaves [101].

Expression analysis of an ash lipoxygenase 3 (LOX3) revealed the highest mRNA levels in Manchurian ash compared to black and green ash (Figure 5H). LOXs are versatile catalysts that participate in various physiological processes and are ubiquitous in nature [102]. In particular, LOXs in higher plants play a vital role in lipid peroxidation processes during plant defense responses and represent precursors for the biosynthesis of jasmonic acid related products, growth and development, senescence and during abiotic stress [102]–[104]. Although it is thought that LOX genes are up-regulated upon insect attack and/or wounding, perhaps the higher constitute levels of LOX3 in Manchurian ash could represent a primed response to *A. planipennis*.

The current study reports a number of transcription factors and other early regulators that are potentially involved in resistance to *A. planipennis.* Further studies need to be performed to learn the molecular functions of these reported genes which were observed to be expressed more abundantly in resistant Manchurian ash compared to the susceptible NA ash species.

### Molecular markers

We have identified 1,272 single nucleotide polymorphisms (SNPs) in 410 ash transcriptome sequences (Table 3 and Table S5). Among them, 823 were transitions, i.e., changes from one purine to another purine or one pyrimidine to another pyrimidine, and 449 were transversions, changes between purines or pyrimidines (Table 3). This ratio of transitions to transversions (2∶1) of SNP occurrence in ash correlates well with other systems [105]. About 94% of the microsatellite loci predicted were dinucleotide (389) and tri-nucleotide repeats (532) followed by quad-nucleotide (37), hexa-nucleotide (17) and penta-nucleotide (5) repeats (Table 4 and Table S6). In general, EST-derived microsatellites are shorter than the genomic microsatellites [106], however, long dinucleotide microsatellites (CT)_22_ were predicted in the current study. Primers were designed for 25 microsatellites (10 primers for dinucleotide repeats and 15 primers for trinucleotide repeats) from the above predicted microsatellites. Seventeen of the 25 primers showed single band amplification in a PCR run, out of which seven were genotyped to check for polymorphism among three ash species (white, green, and Manchurian). Results indicate all seven loci to be polymorphic among the three species studied, providing a valuable resource of molecular markers for ash (Table 5). Similar observations were reported in transcriptomic studies of *C. sativus* and *E. grandis*, wherein 454 pyrosequencing was shown to be an excellent method for large scale prediction of molecular markers for future genetic linkage and QTL analysis in non-model organisms [13], [16]. Given that these microsatellite and SNP markers were predicted from transcriptomic sequences, they are likely linked to protein-coding genes, and therefore might have substantial physiological implications.

**Table 3:** Summary of putative SNPs in *Fraxinus* spp. transcriptomic sequences.

SNP types	Number
Transition	
A-G	411
C-T	412
Transversion	
A-C	123
A-T	137
C-G	80
G-T	109
Total	1,272

**Table 4:** Summary of microsatellite loci predicted in *Fraxinus* spp. transcriptomic sequences.

Number of repeats	Di-nucleotide repeats	Tri-nucleotide repeats	Quad-nucleotide repeats	Penta-nucleotide repeats	Hexa-nucleotide repeats
5		312	22	4	14
6		128	9		3
7		52	2	1	
8	129	14	1		
9	86	10			
10	46	3	1		
11	34	2	2		
12	29	8			
13	10	1			
14	12				
15	8	1			
16	8				
17	3	1			
18	6				
19	3				
20	9				
21	1				
22	2				
23	1				
24	2				
**Subtotal**	389	532	37	5	17

**Table 5:** Number of alleles in seven loci of three ash species (*Fraxinus americana*- White, *F. mandshurica*- Manchurian and *F. pennsylvanica*-Green) respectively.

	Locus						
Species	ASH1502	ASH2429	ASH7867	ASH9764	ASH35207	ASH43402	ASH53476
White	4	7	4	2	1	5	4
Manchurian	2	1	1	1	2	3	1
Green	5	3	1	3	3	4	2

The basic understanding of host resistance mechanisms in angiospermous trees including ash against woodborers is very limited. While NA ash species (green, black and white ash) are highly susceptible to *A. planipennis*, the incidence of attacks against Asian ash species including Manchurian and Chinese has been historically low, suggesting host resistance in the latter [107]. To date there is no evidence that any native NA ash species possesses resistance to EAB attack, which makes the entire ash population highly vulnerable to EAB invasion. Thus, results stemming from this functional genomics study to discover host resistance factors in ash, could feed into future ash breeding/genetic improvement programs.

### Conclusions

The utilization of second generation sequencing for ash species has revealed various metabolic pathways that are of high interest with respect to ash resistance to *A. planipennis*. Data pertaining to the constitutive expression levels of early gene regulators in different ash species, revealed higher levels in Manchurian ash compared to NA ash. Results obtained in this study will lay the foundation for future differential gene expression analysis among different ash species and in deciphering the pathways of secondary metabolism which is related to plant defense. Molecular markers predicted in the current study will further help in population genomics and gene based association studies. These studies will provide critical insights to develop NA ash species that are resistant to *A. planipennis* through breeding programs and/or the application of transgenic technology.

## Materials and Methods

### Sample collection and RNA extraction

Two 10 mm in diameter phloem plugs from un-infested (by *A. planipennis*) green (*F. pennsylvanica*), white (*F. americana*), black (*F. nigra*), blue (*F. quadrangulata*) and Manchurian ash (*F. mandshurica*) were collected in February 2009 from a common garden established in 2003 at Novi, MI. The trees sampled did not have D-shaped exit holes and/or vertical splits on the trunk which are indicators of EAB infestation [3]. At least three different trees were sampled per species and the phloem plugs were immediately wrapped in aluminum foil and stored in liquid nitrogen. Approximately 70 mg of phloem tissue (phloem plugs homogenized in liquid nitrogen) per species was used for RNA extraction. Total RNA was extracted using Trizol® Reagent (Invitrogen, Carlsberg, CA) following manufacturer's protocol and stored at −80°C until further use.

### cDNA library construction

The RNA extracted from the five *Fraxinus* species described above was aliquoted and pooled to construct a cDNA library. RNA isolated from different ash species was pooled in order to capture a diverse population of transcripts and potential species specific transcripts. Further, pooling of the RNA samples represents a cost effective transcriptomic approach to build an EST database for closely related species of a non-model organism. A SMART cDNA library construction kit (Clontech, Mountain View, CA) was used following manufacturer's protocol with modifications: i) A modified CDSIII/3′ primer (5′-TAG AGG CCG AGG CGG CCG ACA TGT TTT GTT TTT TTT TCT TTT TTT TTT VN-3′; PAGE purified) and SuperScript II reverse transcriptase (Invitrogen, Carlsberg, CA) were used for first-strand cDNA synthesis, ii) cDNA size fractionation was excluded and final products were cleaned and eluted using a QIAquick PCR purification kit (Qiagen, Valencia, CA).

### Roche 454 sequencing

cDNA was sheared by nebulization and DNA fragments of approximately 500–800 bp were isolated by agarose gel electrophoresis and subsequent extraction. The isolated DNA was blunt ended, ligated to adapters and immobilized on beads. Single stranded DNA was later isolated from these beads. The isolated library was subjected to Quality Control using RNA 6000 (Agilent Technologies). Concentration and ligation of adapters were estimated using quantitative real-time PCR (qPCR). The emPCR reactions were performed to amplify a single template onto a single sequencing bead. One-quarter of a pico-titer plate was sequenced at the Purdue Genomics Core Facility (West Lafayette, IN) using the GS FLX Titanium chemistry (Roche Diagnostics, Indianapolis, IN).

### Bioinformatic analysis

The 454 transcriptome reads were assembled using Newbler software package (Roche Diagnostics) after the removal of adapter sequences. To achieve better consistency, the contigs and singletons were renamed in the format of “ASH454ONE000001” where “ASH” stands for the ash genus, “454” for 454 sequencing technology, “ONE” for the first trial, and “000001” for an arbitrarily assigned number. The ash transcriptome sequences were annotated by searching against GenBank non-redundant database using the BLASTx algorithm [108]. Also, the sequences were compared to the protein sequences of *A. thaliana* in TAIR9 release from The Arabidopsis Information Resource (http://www.arabidopsis.org/) and *P.trichocarpa* v1.1 (http://genome.jgi-psf.org/Poptr1_1/Poptr1_1.home.html) using BLASTx algorithm. Protein domains were identified by searching against the Pfam database release 24.0 [109] using HMMER v3 program [110]. The Blast2GO software [111], [112] was used to predict the functions of the sequences, assign Gene Ontology terms, and predict the metabolic pathways in Kyoto Encyclopedia of Genes and Genome [113]–[115]. Microsatellite markers were identified using the Msatfinder version 2.0.9 program [116]. SNPs in the library were predicted using gsMapper software (Roche Diagnostics) with an arbitrary criterion of at least 4 reads supporting the consensus or variant.

### Gene mining and quantitative real-time PCR

The ash transcriptome database was mined for genes potentially involved in plant defense. The constitutive gene expression profiles of potential early regulators: CDPK349, CDPK361, MYB, LOX, WRKY and ERF were analyzed using qPCR. cDNA was synthesized from green, black and Manchurian ash using a SuperScript™ First-Strand synthesis kit (Invitrogen, Carlsberg, CA) following manufacturer's protocol. We selected these three ash species to include one genetically close to Manchurian (black ash) and a species distantly related (green ash) which show different levels of susceptibility to *A. planipennis* [5], [6], [117]. Primers were designed using Beacon Designer 7 software (Table S7). The cycling parameters were 95°C for 5 min followed by 39 cycles of 95°C for 10 s and 60°C for 30 s ending with a melting curve analysis (65°C to 95°C in increments of 0.5°C every 5 s) to check for nonspecific product amplification. Relative gene expression was analyzed by the 2^-ΔΔCT^ method [118]. An ash glucose-6-phosphate dehydrogenase (G6PD) was used as the internal reference gene, which has been previously shown to serve as a good internal control in plants [119].

### Microsatellites analysis

Samples from eight individual trees of green, white and Manchurian ash were collected from the U.S. Forest Service, Northern Research station experimental plot Delaware, OH. Genomic DNA was extracted using E.Z.N.A. DNA kit (Omega Bio-Tek, Northcross, GA). Primers were designed for 25 of the predicted microsatellite markers of which only seven were used for genotyping (Table S8). Amplifications were performed in 10 µl reactions. Each reaction contained 5 µl of 2X-Failsafe PCR mix (Epicentre Biotech, Madison, WI), 0.5 U Taq polymerase, 2 pmol reverse primer, 4 pmol modified forward primer (M13 sequence at 5′ end) and ∼10 ng of DNA. M13-tagging protocol was followed using 4pmol of M13 fluorescently-tagged primer (5′-CACGACGTTGTAAAACGAC-3′) [120]. Thermocycling conditions were as follows: 94°C for 5 min, 35 cycles of 94°C for 20 s, 59°C for 20 s, 72°C for 30 s followed by eight cycles of 94°C for 30 s, 53°C for 15 s and 72°C for 30 s with a final extension at 72°C for 10 minutes [121]. PCR products were genotyped using Beckman-Coulter CEQ8800XL (Fullerton, CA) at the Molecular and Cellular Imaging Center (OARDC, Wooster, OH). Alleles were determined using CEQ Fragment Analysis software.

### Data Deposition

The Roche 454 reads of *Fraxinus* species were submitted to NCBI Sequence Read Archive under the accession number of SRA020745.3

## Supporting Information

Table S1 **Comparison of** ***Fraxinus*** **spp. sequences with the model plants** ***Arabidopsis thaliana*** **and** ***Populus trichocarpa.***
(XLSX)

Table S2 **Gene Ontology annotation results of** ***Fraxinus*** **spp. sequences.**
(XLSX)

Table S3 **KEGG summary of** ***Fraxinus*** **spp. sequences.**
(XLSX)

Table S4 **Pfam domain search of** ***Fraxinus*** **spp. sequences.**
(XLSX)

Table S5 **Predicted SNPs in** ***Fraxinus*** **spp. sequences.**
(XLSX)

Table S6 **Microsatellites (SSRs) loci in** ***Fraxinus*** **spp. sequences.**
(XLSX)

Table S7 **List of primers for** ***Fraxinus*** **qPCR analysis**
(DOC)

Table S8 **List of primers for SSRs identified from** ***Fraxinus*** **spp.**
(DOC)
