# Supplementary material for: Transcriptomic Signatures of Ash (Fraxinus spp.) Phloem
Source: PLoS One. 2011 Jan 21;6(1):e16368. doi: 10.1371/journal.pone.0016368 (PMC3025028; doi:10.1371/journal.pone.0016368)
Supplement: Table S7 — List of primers for Fraxinus qPCR analysis (DOC) [file pone.0016368.s007.doc]

**Table S7**: List of primers for *Fraxinus* qPCR analysis.

| **Gene** | **Primer Sequence (5'-3')** | **Primer length (bp)** | **Primer Tm (^o^C)** | **Product size (bp)** |
| --- | --- | --- | --- | --- |
| CDPK349 | GCA ATG ATG AGG AAA GGC AAC C | 22 | 56.9 | 145 |
|  | TCC GCA TAA TCC ACC TCT GTT C | 22 | 56.8 |  |
| CDPK361 | CGG CAG AGT CAA GAG GTC TAT C | 22 | 56.7 | 113 |
|  | AGG AGA ATG CTT GTT CGG GAT C | 22 | 57.1 |  |
| ERF | TCA TCT GTC TGC TCG GCT GTT ATC | 24 | 58.6 | 79 |
|  | ACC TGA GTT TCT TTG CTG GGC TAG | 24 | 59.1 |  |
| G6PD | AGG GCA GGT TAT GTT CAA ACA C | 22 | 55.6 | 117 |
|  | CAC ACG ACC TTA TTG ACA GAG C | 22 | 55.7 |  |
| MYB8679 | GGA TGG GCA GTT TCT GGT GAT G | 22 | 58.3 | 141 |
|  | GAA ACT CGT CGT CTG GAC TCA G | 22 | 58.5 |  |
| LOX3 | CGA GGT ATT CCG AAC AGC ATC TC | 23 | 57.3 | 142 |
|  | ATT TCC CAC AAA TCC ATT CCA CAG | 24 | 55.9 |  |
| MYB10337 | GAG CGA AGA AGA GCA CGA CAA | 21 | 58.1 | 111 |
|  | CAT GAC TGC GAA TCT GGA TGA CTG | 24 | 57.7 |  |
| WRKY7 | GCT GTC TGA AGG TTC TCC ATC TG | 23 | 57.3 | 101 |
|  | CCT GCT TCT GCC CGT ATT TCC | 21 | 58.4 |  |
| WRKY21 | TCT TCA CAC CAC AGG CTG TAC | 21 | 56.9 | 147 |
|  | AGA ACC GAG ATT TGC GTT CAT G | 22 | 55.8 |  |
