# Supplementary material for: Transcriptomic Signatures of Ash (Fraxinus spp.) Phloem
Source: PLoS One. 2011 Jan 21;6(1):e16368. doi: 10.1371/journal.pone.0016368 (PMC3025028; doi:10.1371/journal.pone.0016368)
Supplement: Table S8 — List of primers for SSRs identified from Fraxinus spp. (DOC) [file pone.0016368.s008.doc]

Table S8: List of primers for SSRs identified from *Fraxinus* spp.

| Locus | Forward (5'-3') | Reverse (5'3') | Repeat motif | Size (bp) |
| --- | --- | --- | --- | --- |
| ASH1502 | AAGCGGTTAAGTAAATGACCCA | CGGCACAAAACCAAAACAG | (at)10 | 155 |
| ASH2429 | GACTGGCATTTTTGAGGTCAGTG | CAGGTGGGTTCTGATTCGAT | (ttc)7 | 252 |
| ASH7867 | GACGAGGAGATAATCGGTTGCCA | GCAATGTCGCTTCATATTACAA | (tta)9 | 264 |
| ASH9764 | GACGATGCATTGTCTGACAGGGA | TCCAAACATCCACCCCTTTA | (aag)7 | 252 |
| ASH35207 | GACTTTGGATTCATCCATAGCCA | AAGCAGAACCCAGATCAAGA | (att)7 | 260 |
| ASH43402 | GACTTTGGATTCATCCATAGCCA | AAGCAGAACCCAGATCAAGA | (att)7 | 260 |
| ASH53476 | GACTGATAACTGAAGACACGGCA | AAAGGAACCACAAAGATGCG | (tct)7 | 253 |
